# Comprehensive phenotyping revealed transient startle response reduction and histopathological gadolinium localization to perineuronal nets after gadodiamide administration in rats

**DOI:** 10.1038/s41598-020-79374-z

**Published:** 2020-12-28

**Authors:** Johanna Habermeyer, Janina Boyken, Julia Harrer, Fabio Canneva, Veronika Ratz, Sandra Moceri, Jakob Admard, Nicolas Casadei, Gregor Jost, Tobias Bäuerle, Thomas Frenzel, Christoph Schmitz, Gunnar Schütz, Hubertus Pietsch, Stephan von Hörsten

**Affiliations:** 1 Department of Experimental Therapy, University Hospital Erlangen and Preclinical Experimental Animal Center (PETZ), Friedrich-Alexander-University Erlangen-Nuremberg (FAU), Universitätsklinikum Erlangen (UKEr), Palmsanlage 5, 91054 Erlangen, Germany; 2 Bayer AG, MR & CT Contrast Media Research, Muellerstrasse 178, 13353 Berlin, Germany; 3 Institute of Medical Genetics and Applied Genomics, University of Tuebingen, Tuebingen, Germany; 4 DFG NGS Competence Center Tuebingen, 72076 Tuebingen, Germany; 5 Department of. Radiology, University Hospital Erlangen and Preclinical Imaging Center Erlangen (PIPE), Friedrich-Alexander-University Erlangen-Nuremberg (FAU), Universitätsklinikum Erlangen (UKEr), Erlangen, Germany; 6 Department of Neuroanatomy, Ludwig-Maximilians-University, Munich, Germany

**Keywords:** Sensorimotor processing, Brain imaging

## Abstract

Gadolinium based contrast agents (GBCAs) are widely used in clinical MRI since the mid-1980s. Recently, concerns have been raised that trace amounts of Gadolinium (Gd), detected in brains even long time after GBCA application, may cause yet unrecognized clinical consequences. We therefore assessed the behavioral phenotype, neuro-histopathology, and Gd localization after repeated administration of linear (gadodiamide) or macrocyclic (gadobutrol) GBCA in rats. While most behavioral tests revealed no difference between treatment groups, we observed a transient and reversible decrease of the startle reflex after gadodiamide application. Residual Gd in the lateral cerebellar nucleus was neither associated with a general gene expression pathway deregulation nor with neuronal cell loss, but in gadodiamide-treated rats Gd was associated with the perineuronal net protein aggrecan and segregated to high molecular weight fractions. Our behavioral finding together with Gd distribution and speciation support a substance class difference for Gd presence in the brain after GBCA application.

## Introduction

Gadolinium-based contrast agents (GBCAs) improve magnetic resonance imaging (MRI)-based diagnosis and monitoring of variable conditions and diseases, including but not limited to myocardial infarcts, glioblastomas and multiple sclerosis^1–3^. In 1988, the first MR contrast agent was introduced using the paramagnetic properties of the lanthanide gadolinium (Gd) to shorten the relaxation time of protons in T1-weighted MRIs, thereby enhancing contrast and adding diagnostic value^4,5^.

Eight GBCAs have received marketing authorization, all with favorable safety profiles. To date more than 30 million contrast-enhanced MRI procedures are performed worldwide per year^6^ with numbers continuing to increase.

In 2006, GBCAs were linked to the rare condition nephrogenic systemic fibrosis (NSF), in which renally impaired patients developed symptoms such as skin thickening, fibrosis and lesions after GBCA administration^7^. NSF cases decreased significantly beginning in 2007 when appropriate practices avoiding the use of linear GBCAs in renally impaired patients went into effect.

Clinical studies in healthy volunteers demonstrated that more than 99% of the injected dose of all GBCAs is rapidly eliminated from the body. However, residual traces of Gd were first reported in 1998 in renally impaired patients^8^ and later in 2004 also in patients with normal renal function^9^. In 2014, the group of Kanda et al*.* were the first to report increased signal intensity (SI) in the dentate nucleus and globus pallidus brain areas on unenhanced T1-weighted MRIs of patients without known brain pathologies after repeated administration of the GBCA gadodiamide and gadopentetate dimeglumine^10^. To date, the phenomenon of Gd presence in the brain has been confirmed by retrospective clinical studies. The majority of imaging studies showed that the MRI hyperintensity is only associated with the class of linear GBCAs^11,12^. Until now, an association between the increased SI in the brain after repeated GBCA administrations and clinical adverse events has not been shown.

Based on this increased SI found on unenhanced MR images month to years after the last of multiple administration of linear GBCA^11^ concomitant with the evidence of Gd presence in brain^13^, the European Commission suspended marketing authorization of some linear GBCAs and restricted the use of others. The US Food and Drug Administration (FDA) encouraged further investigation of this phenomenon.

To date, there is an increasing body of evidence that the difference in stability between macrocyclic and linear GBCAs plays a crucial role for the presence of Gd in the brain. In comparison to macrocyclic GBCAs, in which the Gd^3+^ ion is bound in a rigid cage of the chelating ligand forming a highly stable complex, the class of linear GBCAs is less stable since the Gd^3+^ ion is chelated by a poly-aminocarboxylic acid backbone that wraps as an open chain around the Gd^3+^ ion in the center of the complex^14^. While more than 99% of the injected dose of all GBCAs is rapidly eliminated from the body, pre-clinical research found a clear difference for the terminal elimination between linear and macrocyclic GBCAs^15^. These studies demonstrated that macrocyclic agents stay intact and are continuously eliminated from the brain, whereas residual Gd from linear agents was found to be present in different molecular species including a larger molecular weight Gd species^16,17^, indicating a possible interaction of Gd with a biological molecule with unknown functional consequences. De-chelation might occur through transmetallation, which implies the displacement of Gd^3+^ ions from the ligand by other metal ions (for example zinc ions or copper ions^18^) allowing Gd^3+^ ions to bind to an anionic partner. Thus, while this process is highly dependent on the kinetic stability of the GBCA, and likely to only occur with linear chelates^19,20^, it is still not fully understood which molecular Gd species are present in the brain. Since Gd can still be found years after the last administration of linear GBCAs, it is very likely that the Gd^3+^ ion has left its chelate by immediately binding to other endogenous molecules or macromolecular structures in the brain^21^. Potential new binding partners for the released Gd^3+^ ions are anions resulting in the precipitation of Gd-salts such as GdPO_4_ or Gd_2_CO_3_ or negatively charged endogenous macromolecules^22^.

The question whether potential clinical consequences are induced by such a Gd-marcomolecule interaction or the GBCAs itself is difficult to answer retrospectively, since GBCAs are clinically used in the context of severe disease conditions with pre-existing symptoms and are rarely administered to healthy humans. Furthermore, the onset of potential Gd-induced symptoms could occur years after the last GBCA exposure or could depend on a certain threshold of Gd in tissue reached after multiple administrations of GBCA over time. In addition, the lack of continuous documentation of patients’ clinical history and missing comparability between study cohorts impedes investigation of long-term effects of GBCA administration and Gd residuals in humans.

In this study we therefore investigated potential consequences of residual Gd present in rat brains after repeated administration of body surface adjusted high clinical doses of the linear gadodiamide and macrocyclic gadobutrol (representing the two marketed GBCA classes), by comprehensively and longitudinally assessing the behavioral and neuro-histopathology phenotype.

## Results

**Figure 1:**
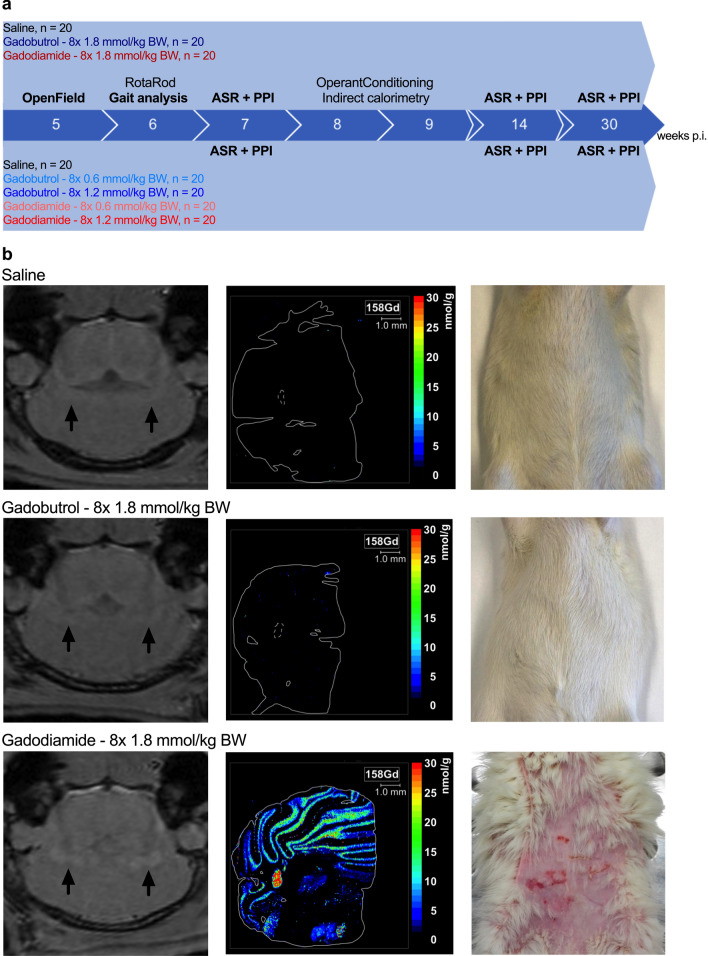
Comprehensive and longitudinal behavioral phenotyping after repeated administration of clinical doses of linear gadodiamide and macrocyclic gadobutrol in a rat model. (**a**) Experimental setup for longitudinal and comprehensive behavioral phenotyping from 5 to 30 weeks after the last injection showing time points of the behavioral tests. (**b**) Representative images from T1-weighted (T1w) MRI at 4.7 T in axial orientation 5 weeks p.i. Regions of interest in the lateral cerebellar nuclei are marked by arrows (left panel). Spatial distribution and quantification of Gd in the cerebellum at 11 weeks p.i. (middle panel). Macroscopic NSF-like skin lesions in gadodiamide rats observed until 12 weeks p.i. (right panel).

This study is based on a comprehensive behavioral testing of Wistar rats for up to 30 weeks after repeated injection of linear gadodiamide or macrocyclic gadobutrol, representing the two marketed GBCA classes, at 8 × 0.6, 8 × 1.2 or 8 × 1.8 mmol/kg BW (Fig. 1a).

### Animal model confirmed increased SI and the presence of residual Gd in the brain after multiple administrations of the linear GBCA gadodiamide

MRI performed 5 weeks p.i. revealed visually evident enhanced deep cerebellar nuclei (DCN) after administration of gadodiamide at 8 × 1.8 mmol/kg BW (Fig. 1b, left panel). The respective quantitative DCN/brain-stem SI ratio was significantly higher (1-way ANOVA with post-hoc Dunnett´s multiple comparison test: *p* < 0.001) for linear gadodiamide (1.06 ± 0.03) compared to the macrocyclic gadobutrol (1.00 ± 0.02) or saline (0.99 ± 0.03). Gd presence in the brain was confirmed using laser-ablation-inductively-coupled plasma-mass spectrometry (LA-ICP-MS; Fig. 1b, middle panel). In line with published preclinical and clinical data, Gd was found in the lateral cerebellar nucleus in animals that received 8 × 1.8 mmol/kg BW gadodiamide but not gadobutrol 11 weeks p.i. (mean Gd concentration 37.8 ± 5.7 nmol/g versus 0.12 ± 0.05 nmol/g; Supplementary Fig. 3a, representing about 0.0013% and 0.000004% of the injected dose per gram tissue, respectively).

We observed that 11 out of 20 rats treated with 8 × 1.8 mmol/kg BW gadodiamide developed NSF-like cutaneous symptoms, which were not observed in gadobutrol and control animals (Fig. 1b, right panel). The affected rats showed extensive dorsal and rostral loss of fur and macroscopic skin lesions.

### General activity, emotional status and gait were not affected

The following behavioral tests were conducted (Fig. 1a, Table 1): Open field test, RotaRod, gait analysis (CatWalk XT system), acoustic startle reaction (ASR) and pre-pulse inhibition (PPI) testing, as well as assessment of cognitive (operant conditioning paradigm) and metabolic status (indirect calorimetry).

**Table 1:** Behavioral domains addressed, and respective behavioral tests performed.

Domain	Assay	Effect of gadobutrol	Effect of gadodiamide
General health	SHIRPA	−	−
Emotionality	OpenField	−	( +)
Locomotion	Gait analysis*	−	−
	RotaRod	−	−
Sensorimotorfunction	Acoustic Startle Reaction	−	+
	Prepulse Inhibition	−	−
Metabolism	Indirect calorimetry**	−	−
Cognition	Operant Conditioning	−	−

Observed effects are indicated by -: no effect observed; (+): effect observed, potentially confounded; + : effect observed (no confounding condition). *using the CatWalk XT system, **measured with the PhenoMaster setup.

Bodyweight (BW) of the animals was measured at least twice per week and was not affected by GBCA administration (Supplementary Fig. 1h). Key features of the metabolic analysis by indirect calorimetry, such as food/water intake and respiratory exchange rate were not altered by GBCA administration (Supplementary Fig. 2a–c). Cognitive testing revealed no differences between control and GBCA groups (Supplementary Fig. 2d).

**Figure 2:**
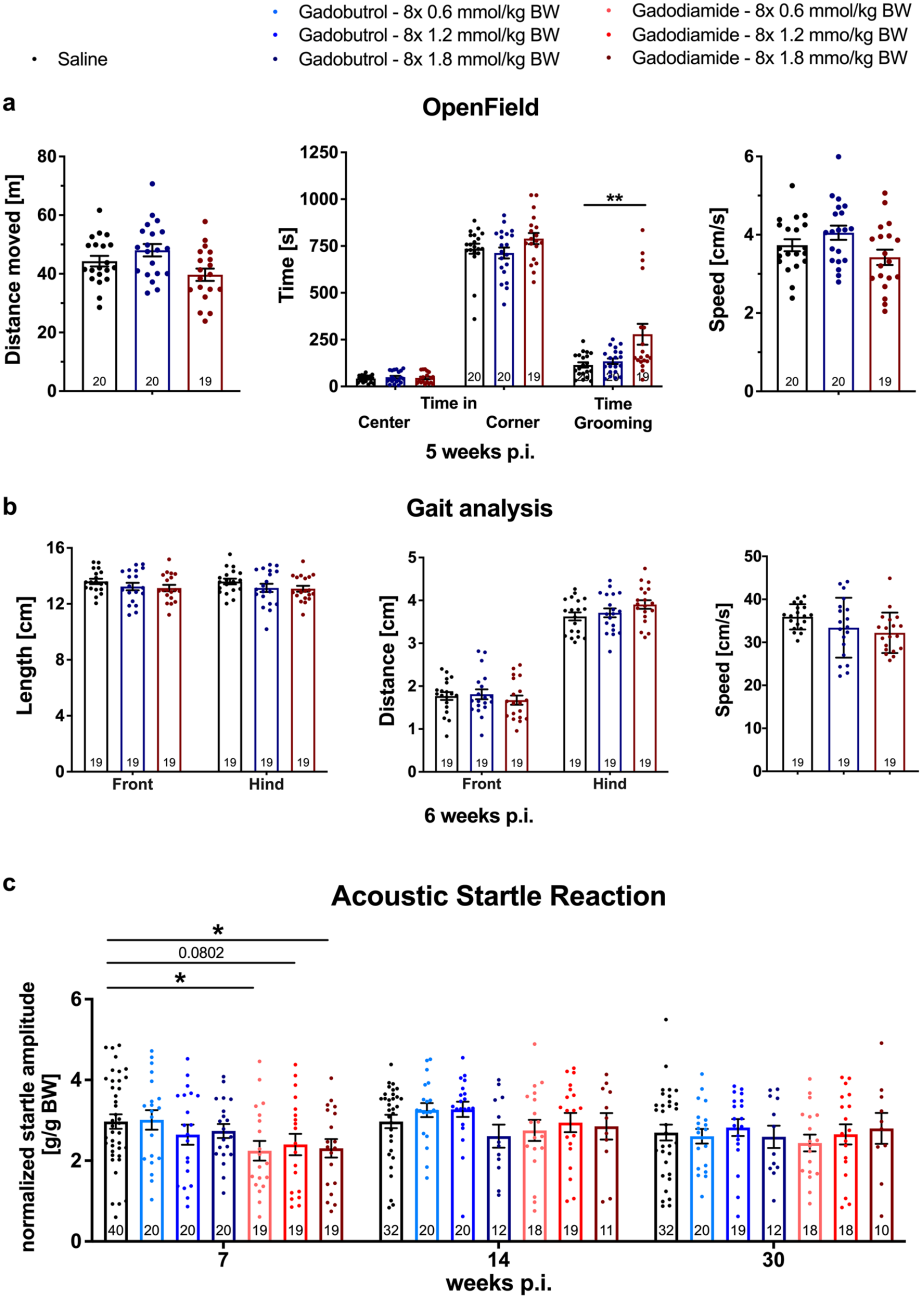
Early and transient reduction in acoustic startle reaction in gadodiamide-treated animals and comparable parameters in the OpenField test and gait analysis. (**a**) Explorative and grooming behavior 5 weeks p.i in the OpenField test. (**b**) Stride length of front and hind paws, distance between either front or hind paws (base of support) and average speed of the animals measured with the CatWalk XT system test 6 weeks p.i. (**c**) Startle reaction to 120 dB acoustic stimuli 7, 14 and 30 weeks p.i. Data represent mean ± SEM; n is indicated in the columns; *p < 0.05; **p < 0.01.

To assess general activity, emotional status and reaction to a novel environment, rats were tested in the open field (Fig. 2a). 1-way ANOVA indicated a significant effect of the factor “GBCA administration” on the distance moved (F_2,56 _= 4.263, *p* = 0.0189), due to the opposite trends of gadobutrol- (increased distance moved compared to saline) and gadodiamide-administered animals (reduced distance moved compared to saline). Subsequent Dunnett's multiple comparisons test revealed no significant alterations of the behavior of treated-animals compared to the control (saline vs. gadobutrol: *p* = 0.3189; saline vs. gadodiamide: *p* = 0.1968). No effect of the GBCA administration on the speed (1-way ANOVA: F_2,56 _= 3.112, *p* = 0.0523) and the preferential position (1-way ANOVA: center: F_2,56 _= 0.3711, *p* = 0.6917; corner: F_2,56 _= 1.890, *p* = 0.1606) was observed. All animals spent more time in the corners than in the center area of the open field.

11 out of 20 animals in the 1.8 mmol/kg BW gadodiamide group showed NSF-like skin lesions (Fig. 1b, right panel). Time spent grooming was prolonged for these animals compared to the saline control (F_2,56 _= 7.134, *p* = 0.0017), which is most likely related to the skin lesions. A specific analysis excluding the affected animals showed no abnormal grooming behavior of gadodiamide animals without NSF-like skin lesions (Supplementary Fig. 1g).

Footfall and motor performance were assessed using the automated CatWalk XT system (Fig. 2b). One animal of each group had to be excluded from the analysis due to strict criteria excluding the influence of factors unrelated to motor functions. The “stride length” (distance between two successive placements of one paw) was neither altered by the factor “GBCA administration” (2-way ANOVA: F_2,108_ = 2.538, *p* = 0.0837) nor was it discrete for front and hind paws (2-way ANOVA: F_1,108_ = 0.0688, *p* = 0.7935). Animals of all groups showed a physiological shorter distance between the front than between the hind paws (“base of support”; 2-way ANOVA: F_1,108_ = 569.5, *p* < 0.0001), while there were no inter-group differences observed (2-way ANOVA: F_2,108_ = 0.4055, *p* = 0.6677). The average speed of the animals on the walkway was not influenced by GBCA administration (1-way ANOVA: F_2,54_ = 2.603, *p* = 0.0834).

The analysis of the motor function by RotaRod was also without significant changes (Supplementary Fig. 2e).

### Transient startle response reduction observed in gadodiamide treated rats

To investigate the basal sensorimotor coupling the animals were tested for their startle reaction to acoustic stimuli at 7, 14 and 30 weeks p.i. Figure 2c depicts the reaction to 120 dB pulses. The full data set including all applied startle intensities and corresponding statistical approach and analysis is given in the supplements (Supplementary Fig. 1a–c). The factor “GBCA administration” (gadobutrol or gadodiamide) had a significant effect (2-way ANOVA: F_6,750_ = 4.797, *p* < 0.0001) 7 weeks after the last injection, referred to the reduced startle reaction of gadodiamide administered animals (Fig. 2c). Startle reactions of animals injected with 8 × 0.6 mmol/kg BW (post-hoc Dunnett´s multiple comparison test split per pulse intensity: p = 0.0117) and 8 × 1.8 mmol/kg BW gadodiamide (post-hoc Dunnett´s multiple comparison test split per pulse intensity: *p* = 0.0266) were significantly reduced, while animals injected with 8 × 1.2 mmol/kg BW showed reduced responses with a *p* = 0.0802 compared to the saline group. By repeating the ASR test at 14 and 30 weeks p.i. no effect of the factor “GBCA administration” was observed (3-way ANOVA: 14 weeks p.i.: F_1,812_ = 0.946, *p* = 0.331; 30 weeks p.i.: F_1,797_ = 0.024, *p* = 0.878). In contrast, no significant alteration of ASR was observed in the gadobutrol group at all investigated time points and doses.

For both GBCA classes, linear and macrocyclic, sensorimotor gating of the animals was not altered (tested in a pre-pulse inhibition (PPI) task; see Supplementary Fig. 1d–f).

### Multiple GBCA administrations showed no effect on the number of neurons or general pathway deregulation in DCN

**Figure 3:**
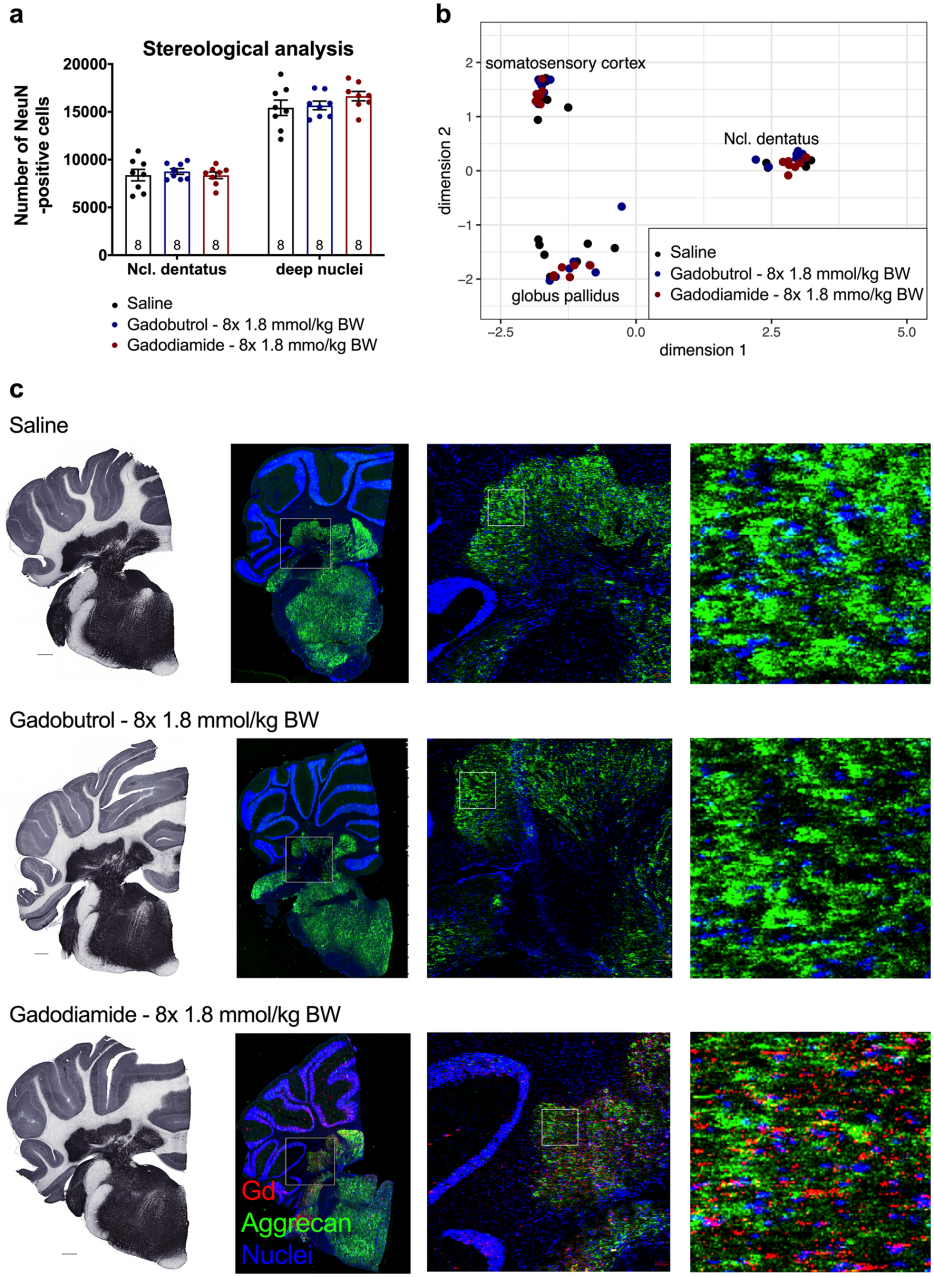
No alterations in number of NeuN-positive cells and gene expression as well as overlap of aggrecan and Gd distribution in the lateral cerebellar nucleus. (**a**) Numbers of NeuN-positive cells in the lateral cerebellar nucleus and adjacent deep cerebellar nuclei of treated and control animals 11 weeks p.i. determined by stereological analysis. (**b**) Gene expression pathway analysis in the lateral cerebellar nucleus, globus pallidus and somatosensory cortex (control region) 11 weeks p.i. (**c**) Conventional immunohistochemistry of aggrecan in the cerebellum of animals sacrificed 11 weeks p.i. (left panel, scale bar: 500 µm). Distribution of Gd and aggrecan in the cerebellum of animals sacrificed 5 weeks p.i. by immunohistological LA-ICP-MS (right panel). Representative pseudo-colored overlays of Gd (red), aggrecan (green) and an iridium containing nucleic acid intercalator (blue) at different magnification. Fields of higher magnification are indicated by squares. Data represent mean ± SEM; n is indicated in the columns.

The observed behavioral effect seen with linear gadodiamide was not associated with a loss of neurons in the brain areas of high Gd presence. Stereological quantification of the number of NeuN-positive cells in the lateral cerebellar nucleus and adjacent DCN revealed no significant difference in the total number of neurons in both GBCA groups (2-way ANOVA: F_2,42_ = 0.6472, *p* = 0.5287; Fig. 3a). Moreover, a hypothesis-free strategy was used to analyze the gene expression profile of the DCN (Fig. 3b showing the most impactful components of the multidimensional scaling analysis). No general pathway deregulation in the DCN of treated animals was identified resulting in overlapping distribution of the groups for each analyzed region.

### Gd brain distribution in gadodiamide administered animals partially overlaps with aggrecan immune staining

We further investigated the perineuronal nets (PNN) as possible interaction partner for released Gd due to their buffer capacity and ability to bind metal ions. The overall protein expression of aggrecan, a proteoglycan of the PNN, in full brain homogenates obtained 5 weeks p.i. was comparable between all groups (Supplementary Fig. 3b). Next, we evaluated the protein expression of aggrecan in coronal sections of one brain hemisphere in the DCN by classic immunohistochemistry (Fig. 3c, left panel). The spatial expression of aggrecan was region specific and the most intense staining occurred in the DCN, the cochlear nuclei and the brain stem. To examine the PNN distribution in comparison to the Gd distribution, LA-ICP-MS was combined with immunohistochemistry by using Maxpar metal tagged secondary antibodies at 5 weeks p.i. The staining pattern for aggrecan was comparable in all animals (Fig. 3c, two middle panels). Only Gd traces were observed for gadobutrol animals, indicating washout of the highly water soluble intact gadobutrol chelate during the experimental procedure. However, the region-specific accumulation of Gd in gadodiamide animals persisted, indicating that the remaining Gd is most likely tissue bound or insoluble precipitate. Overlays of false colored images of the isotopes ^158^Gd, ^175^Lu (aggrecan) and ^193^Ir (DNA intercalator) revealed a spatial correlation of Gd (gadodiamide) and aggrecan in the area of the DCN and in the cochlear nuclei, but not in other regions. High resolution LA-ICP-MS (2µm^2^ spot size) identified an inhomogeneous Gd distribution with small Gd spots and larger Gd clusters within the cerebellar nuclei of gadodiamide animals (Fig. 3c, right panel). Indeed, the Gd distribution (white square, higher magnification) showed partial co-localization with the PNN structure, whereas the nuclei pattern showed only minor overlap with Gd. Nonetheless, not all Gd co-localized with these structures indicating other additional Gd species or binding partners in the gadodiamide group.

### Residual Gd segregates to high molecular weight fraction in the brain

**Figure 4:**
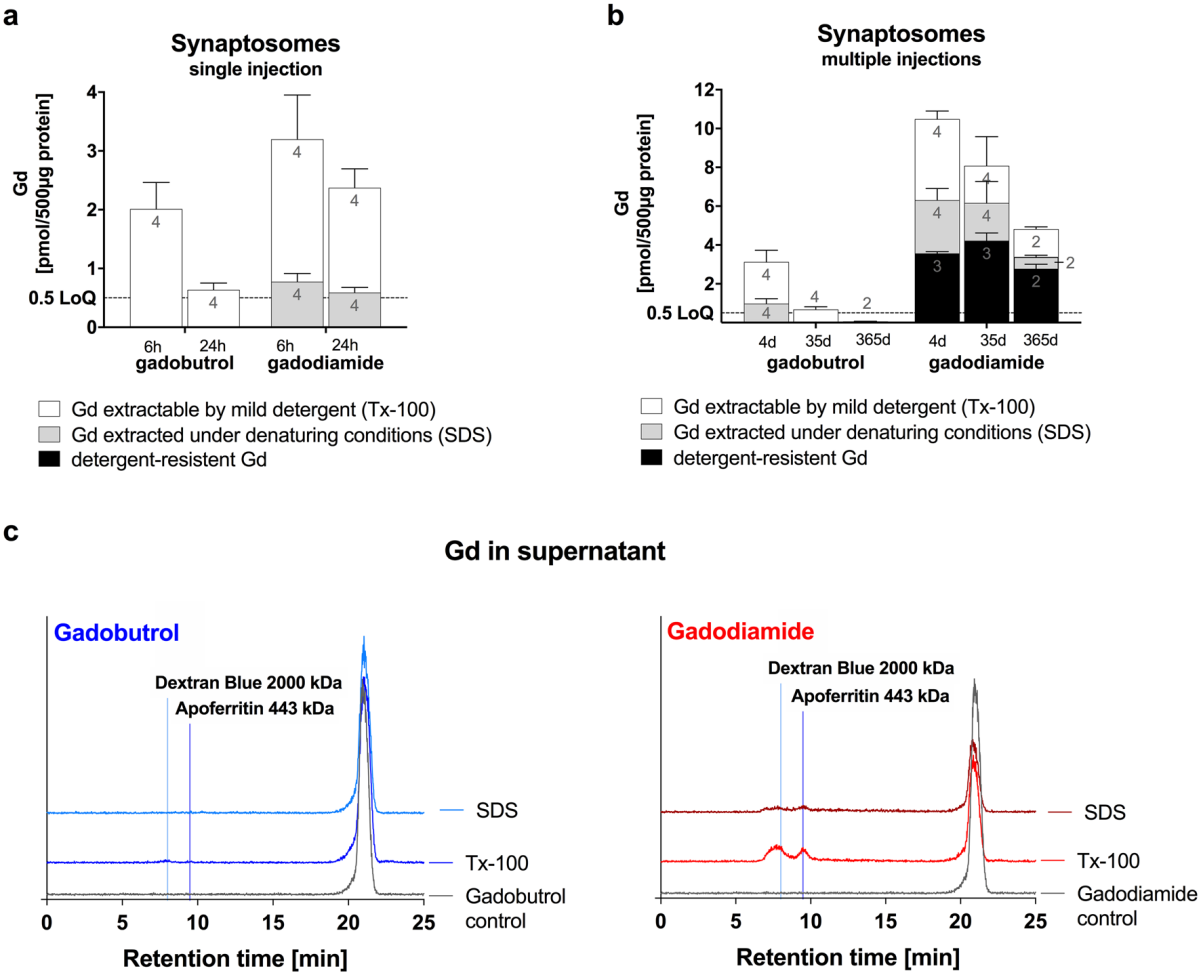
Detergent extraction of synaptosomes purified from GBCA treated animals. Synaptosomes were incubated either with 2% Triton X-100 or 0.5% SDS or detergent free buffer as a control and the resulting synaptosomal pellets were analyzed for Gd by ICP-MS. (**a**) Synaptosomal Gd in animals which received a single injection of GBCA (gadobutrol or gadodiamide, 1.8 mmol/kg BW) and were sacrificed after 6 and 24 h p.i. (**b**) Synaptosomal Gd in animals which received multiple injections of GBCA (gadobutrol or gadodiamide, 8 × 1.8 mmol/kg BW) and were sacrificed after 4, 35 and 365 days p.i. (**c**) The released Gd species in the extracts from synaptosomes of animals that received multiple injections of GBCA (4 days p.i.) were characterized by size exclusion chromatography (SEC)-ICP-MS. Chromatograms of extracted Gd species in comparison to a pure solution of gadobutrol (left panel) and gadodiamide (right panel). Note that for gadodiamide but not for gadobutrol injected animals additional Gd species with large molecular weight (shorter retention time) were observed; Data represent mean ± SD; n is indicated in the columns.

To further address neuronal localization, we evaluated the concentration and distribution of Gd from crude synaptosomes extracted with either the non-denaturing detergent Triton X-100 (Tx-100) or under denaturing conditions using SDS (Fig. 4). In control synaptosomes treated with buffer instead of detergent, the Gd concentration was lower after the administration of macrocyclic gadobutrol compared to the linear GBCA gadodiamide at all time points analyzed. Moreover, the clearance of Gd from synaptosomes over time was superior for gadobutrol compared to gadodiamide. In the early elimination phase (24 h vs 6 h after 1 × 1.8 mmol Gd/kg), the synaptosomal Gd concentration was reduced by 68% in gadobutrol animals, while only a 26% reduction was evident in gadodiamide animals. The slower Gd elimination from synaptosomes in the gadodiamide group was accompanied by the presence of a Gd species that required denaturing conditions for extraction. In contrast, mild solubilization of membranes by Tx-100 was sufficient to release all Gd found in synaptosomes of gadobutrol animals (Fig. 4a).

Due to the fact that not all Gd/GBCA is cleared within 24 h, the multiple daily dosing regimen (8 × 1.8 mmol Gd/kg bw) resulted in a buildup of Gd/GBCA in the synaptosomal fraction 4 days after the last injection (Fig. 4b). In gadobutrol animals, clearance was efficient (− 78% at 35 days p.i.) and complete (below LoQ at 365 days p.i.). In contrast, the clearance was slow (− 23% at 35 days p.i.) and incomplete after one year (− 54% versus 4 days p.i.) in gadodiamide animals. A detergent-insoluble species was observed in gadodiamide animals, which most likely represents inorganic Gd salts. Insoluble Gd species were not present in gadobutrol animals.

Size exclusion chromatography was used to further analyze the Gd species present in the detergent extracts of synaptosomes (Fig. 4c). In gadobutrol animals, one Gd species was observed and the retention time of the extracted Gd species was equivalent to the intact Gd chelate. In contrast, three Gd species were found in the synaptosomal extracts after mild solubilization with Tx-100 in gadodiamide animals. In addition to the intact Gd chelate, a large molecular weight species with a retention time comparable to apoferritin (corresponding to a molecular size of approximately 440 kDa) as well as an ultra large species that eluted with the void volume marker (≥ 2000 kDa) were observed after extraction with Tx-100. These high molecular weight species were destroyed under denaturing conditions indicating a proteinaceous molecule.

## Discussion

In this study, we investigated potential effects of GBCA-associated hyperintensities on T1-weighted MRI and residual Gd in the brain after multiple administrations of linear and macrocyclic GBCAs.

Most clinical studies addressing the presence of Gd in brain are either case reports or retrospective studies describing small cohorts of a few patients. They are limited by the lack of sufficient controls for confounding factors, including prior use of other GBCAs, different time-points of Gd measurement, varying GBCA doses and time intervals between GBCA administration and tissue sampling. For that reason, most studies investigating Gd presence in the brain were performed in animals. The majority of these studies focused on effects of GBCA or other forms of Gd in models reflecting a certain disease status as for example blood–brain-barrier disruption and renal impairment^21–25^ or mimicking sub-cohorts like pregnant women or pediatric patients^26,27^. Our literature survey revealed only a handful of preclinical studies, also including behavioral assays to test for potential clinically relevant consequences of Gd presence^24,24–28^. To our knowledge, this is the first study extensively screening for behavioral abnormalities associated with GBCAs in a comprehensive and longitudinal manner, covering a broad range of behavioral domains including motor function, emotional and metabolic status and sensorimotor function in a well-established rat model with excellent face validity^29^. As expected, rats receiving gadodiamide showed increased SIs on T1-weighted MRI in the DCN. Measurement of the Gd concentration in the cerebellar nuclei at 11 weeks p.i. confirmed the presence of Gd in gadodiamide administered animals but only minor residual Gd in rats that received gadobutrol.

We found NSF-like cutaneous symptoms^30^ in rats treated with the highest dose of 1.8 mmol/kg BW gadodiamide, which was a confounding factor for one behavioral test: The dermatologic abnormalities associated with gadodiamide led to prominent grooming behavior of these animals in the Open field test. A study of Cacheris et al. from 1990 describes hair loss, skin lesions, gastric and cutaneous mineralization and subacute inflammation in rats receiving 5.0 mmol/kg BW gadodiamide^31^.

We observed a dose-dependent and transient reduction of startle reaction in all groups of animals administered with the linear GBCA gadodiamide 7 weeks p.i. This effect was not observed in rats that received the macrocyclic GBCA gadobutrol, indicating that the effect might be associated with released Gd from the less stable gadodiamide. Startle reactions of gadodiamide-treated animals were reduced by 20% compared to the control, describing a rather mild impairment of sensorimotor function^32^. The primary startle pathway consists of the auditory nerve, the ventral cochlear nucleus, the dorsal nucleus of the lateral lemniscus, the caudal pontine reticular nucleus, spinal interneurons and spinal motor neurons^32,33^. Since no hearing deficits were observed during primary screening of the animals and startle responses appeared to be normal 14 weeks p.i., we assume that the ASR circuit is not irreversibly affected. Surprisingly, the modulation of the startle reaction by acoustic pre-stimuli (PPI) was not altered. This superordinate inhibitory circuitry modulating the ASR involves several neurotransmitters and brain regions, including the inferior and superior colliculus and pedunculopontine tegmental nucleus^34^. PPI is given as the percentage of decline in the startle response (formula: see Method-section), meaning the startle of the pre-pulse trial is normalized to the baseline startle amplitude of each individual animal. Therefore, a generally reduced ASR is not represented by the PPI measurement. Interestingly, similar findings with reduced ASR and normal PPI were published for rats with iron deficiency, which is suggested to alter dopamine metabolism^32–36^.

To reveal potential molecular and/or cellular patho-mechanisms underlying our findings in behavioral tests we used a hypothesis-free strategy and analyzed the gene expression profile of the DCN. We could not identify a general pathway deregulation in the DCN of treated animals. Based on our data, we cannot exclude a change in gene expression in nuclei of the auditory circuit affecting the ASR in gadodiamide-administered animals. Examination of the spatial Gd distribution by LA-ICP-MS revealed increased Gd levels not only in the DCN but also in the cochlear root nucleus, which is involved in the auditory circuitry. Nevertheless, normal gene expression profiles in the DCN may reflect the current body of knowledge since no clinical symptoms could be ascribed to Gd presence in the brain weeks or months after the last GBCA injection^37,38^.

GBCAs are Gd-chelates, shielding the Gd^3+^ ion from contact with biomolecules. A well-known feature of Gd^3+^ ions is its cytotoxicity in vitro and in vivo when applied in the unchelated form, causing cell death in liver, kidneys and neural tissue presumably by binding to endogenous interaction partners^28,37–41^. The (neuro-)toxic property of Gd^3+^ ions is partly based on its size similar to the calcium ion (Ca^2+^) resulting in competitive inhibition of Ca-dependent biological processes^42,43^. Gd^3+^ ions have also been shown to trigger apoptosis and induce elevated reactive oxygen species in rat cortical neurons^41^. Based on these data we examined the number of NeuN-positive cells in the lateral cerebellar nucleus and adjacent DCN. No neuronal loss was found in brain specimens collected 11 weeks after the last injection. This is consistent with the human autopsy study by Fingerhut et al*.*, who observed no neuronal loss in patients who received serial injections of GBCA^44^.

We further evaluated potential mechanisms that might play a role in the behavioral phenotype. Since synaptic transmission mediates the communication between nerve cells, we applied a subcellular fractionation of rat brain to investigate potential accumulation and clearance of GBCA from synaptosomes. We observed small amounts of Gd in synaptosomes with substantial differences for the linear gadodiamide in both, amount and extractability, compared to the macrocyclic gadobutrol. Even though we cannot completely rule out that part of the synaptosomal Gd has been artificially incorporated during sample processing, the differences in the amounts and time points (in total and as percentage of Gd in brain homogenates) indicate specific co-enrichment.

Although the analyzed GBCA cannot actively enter cells, passive mechanisms such as endocytosis raise the possibility that components of the extracellular fluid are taken up in an unspecific manner, including small amounts of GBCA. To maintain proper brain function, endocytic recycling of synaptic vesicle membrane together with incorporated proteins is necessary to balance membrane surface area and to ensure efficient neurotransmission^45,46^. A study by Shi et al*.* showed that neuronal excitation influences the transportation speed of the GBCA in the brain with prolonged presence after stimulation^47^.

One open question is whether the Gd found in neural tissue is retained in its chelated form or whether the lanthanide was released from the Gd-chelate-complex. In a study by Frenzel et al*.* chromatographic analysis of total rat brain homogenates showed exclusively the intact Gd-chelate-complex in the soluble fraction after gadobutrol administration^21^. In animals that received gadodiamide, in addition to the intact GBCA, Gd was also found bound to macromolecules which is assumed to be responsible for increased SI in MRI, based on the potentially increased rotational correlation time of the newly formed Gd-species. Moreover, an insoluble Gd species was detected, presumably GdPO_4_, which was not found after gadobutrol administration^21^.

In our study, we only detected the intact Gd chelate in synaptosomes for macrocyclic gadobutrol. For linear gadodiamide, we observed a macromolecular Gd species as described above as well as detergent resistant insoluble Gd species. If small amounts of GBCA are indeed taken up by vesicle endocytosis, the stability of the contrast agent most likely impacts the fate of the GBCA. Lowering the luminal pH is essential for synaptic vesicle recycling^47,48^. Glutamatergic synaptic vesicles have been shown to exhibit a resting pH of 5.8, while GABAergic vesicles were found to have a resting pH of 6.4. Interestingly, GABAergic vesicles undergo an initial over-acidification and subsequent alkalization process to restore the resting pH following endocytosis^49^. This acidic environment could promote the dissociation of Gd from the linear chelate, which have a lower kinetic stability. Macrocyclic GBCA are kinetically inert and less vulnerable to low pH and, thus, are released as intact GBCA to the interstitial system and drained from the brain via the glymphatic system^50^.

Based on the current research, our data indicate that released Gd from linear GBCA binds to not yet identified macromolecules occurring in the cerebellar nuclei, which are most probable negatively charged and enrich in the SDS-fraction of synaptosomes. We propose proteoglycans of the extracellular matrix (ECM) as possible binding partners. Taupitz et al. suggested competitive chelation with components of the ECM playing a role in the tissue distribution and toxicity of GBCA earlier^51^. Aggrecan, a highly anionic proteoglycan of the PNN, represents a suitable complexation-partner for Gd: it would preserve the paramagnetic properties, resulting in MRI-hyperintensities; it has an ion buffering capacity to absorb excess cation changes around synapses^52^; it is highly anionic endowed by sulfated glycosaminoglycan chains^53^. Immuno-histological staining for aggrecan as well as the combination of metal-tagged immune-staining and LA-ICP-MS revealed a partially overlapping distribution and co-occurrence of Gd and aggrecan in the cerebellar nuclei. Moreover, studies have shown that proteins of the PNN enrich in synaptosomes during rat brain fractionation^54^ and that denaturing conditions are required for proteoglycan extraction^55,56^. Therefore, the amount of Gd from linear GBCA that is released from synaptosomes after SDS treatment might represent Gd and/or GBCA associated with PNNs. Interestingly, several studies using mice deficient for different PNN-molecules or enzymes important for their synthesis reported altered ASR in these animals^57,58^, giving another hint to a potential interaction of Gd and PNNs with impact on behavior.

In summary, our findings reveal new preclinical insights into the clinically observed phenomenon of Gd presence in the brain. Since many behavioral results are difficult to translate to humans, the relevance of our data has to be investigated in patients.

Reduction of Gd levels in the brain can be observed for up to 52 weeks after administration for macrocyclic GBCA^29^. Therefore, the time point for analytical or behavioral assessments needs to be chosen carefully. Regarding the observed alteration of ASR, earlier testing will be of interest to evaluate the onset of the altered reflex-behavior. Additional testing of different reflex-inducing stimuli can narrow down the underlying responsible brain nuclei. Here it is also of great interest to evaluate the effect of GBCA on the different segments of the auditory system including the more peripheral-located end-terminals of the auditory nerve within the cochlea. Not only the number but also the morphology of the neuronal cells could be evaluated similar to a recently published study, which investigated the density and axonal morphology of intraepidermal nerval fibers^59^. The data also indicate that future research in the direction of extracellular macromolecules as suitable binding partners for de-chelated Gd or GBCA will contribute to the understanding of Gd presence in the brain.

As currently no clinical phenotypes are observed after administration of GBCA in routine clinical practice, we chose a high GBCA dosing to investigate potential effects. The applied triple dose, which was multiplied by 6 on the basis of body surface area adaption guidelines^60^, and the daily dosing regimen for two weeks resulted in a higher GBCA burden as in clinical routine.

The majority of behavioral tests performed in rats after administration of high and repeated doses of macrocyclic and linear GBCA showed no long-term effects. However, a transient and reversible decrease in startle reflex was observed with the linear GBCA gadodiamide, but not with the macrocyclic gadobutrol. These results suggest that further research in the areas of motor reflexes and PNN-GBCA interactions is required to unravel potential biological effects of trace amounts of Gd released from linear GBCAs.

## Methods

### Study design

The study was designed to screen for potential behavioral abnormalities associated with the repeated administration of eight doses (8 × 0.6, 1.2 or 1.8 mmol/kg BW) of gadobutrol or gadodiamide in comparison to a saline control group in a longitudinal (30 weeks p.i.) and comprehensive manner. The following behavioral tests were conducted according to the schedule shown in Fig. 1a: OpenField test, RotaRod (motor function), gait analysis using the CatWalk XT system, investigation of sensorimotor function and gaiting by acoustic startle reaction (ASR) and pre-pulse inhibition (PPI) testing, as well as assessment of cognitive and metabolic status of the animals using an operant conditioning paradigm and indirect calorimetry. Method descriptions and results of the tests not described in the main text are given in the supplementary information.

The study was conducted at the Dept. Experimental Therapy, University Hospital Erlangen, in accordance with the German guideline for animal experiments and was approved by the government of Lower Franconia (DMS2532-2-397), Bavaria, Germany. All experiments were conducted according to local, NIH, and ARRIVE guidelines^61^.

### Animals and doses

Male Wistar out-bred rats (obtained from Charles River, Sulzfeld—Germany) were repeatedly injected intravenously on four consecutive days per week for two weeks either with saline (n = 40) or with the GBCA gadobutrol or gadodiamide at three different body surface adjusted doses of either 0.6, 1.2 or 1.8 mmol/kg BW, representing the recommended clinical single-, double- or triple dose (n = 20 for each dose and GBCA).

The same dosing regimen (8 × 1.8 mmol/kg BW or saline) was applied for the subcellular fractionation experiments. Rats (n = 6 per group and time point) were sacrificed at day 4, 5 weeks and after 1 year. For the subcellular fractionation of brain tissue early after GBCA administration, rats received a single dose of 1.8 mmol/kg BW or saline and were sacrificed 6 h and 24 h after GBCA injection.

### Behavioral assays

ASR was measured according to a standardized protocol described before^62^. Each rat was placed in a small aluminum box, where major movements and exploratory behavior was restricted, and which was tightly anchored to a piezo-accelerometer, positioned in between two loudspeakers inside of a sound-attenuating chamber (TSE Systems GmbH, Bad Homburg—Germany). Experimental protocols started with a 4 min habituation phase, during which a constant background white noise (68 dB) was played and baseline movements of the animals were recorded. The experimental phase followed right after, consisting of 50 test trials 100 ms long, where 20 ms white noise stimuli were played at 75, 95, 105, 115 and 120 dB (10 repetitions each). Noise stimuli were presented in a randomized order with an inter-trial interval of 6–12 s. Startle reaction was normalized to the BW of each individual animal.

Sensorimotor gating was evaluated in a PPI test using the same experimental setup and equipment as described for the ASR test and followed a well-established protocol^62^. The experiment consisted of 77 trials. At the beginning 10 “startle trials” consisting of a single 120 dB pulses (20 ms) were delivered, followed by 40 “PPI trials”, where a pre-pulse noise of 72, 76, 80 or 84 dB (20 ms, 10 repetitions each) was played 100 ms before the startling stimulus (120 dB, 20 ms). The PPI trials were administered in a randomized order, interspersed with 15 additional “startle trials” (120 dB, 20 ms) without presenting a pre-pulse, and 12 additional “pre-pulse control trials”, where each of the pre-pulse stimuli used in the PPI trials was played alone (20 ms, 3 repetitions each). As standard, a 6–12 s randomized inter-trial interval separated the trials throughout the experiment, which lasted approximately 14 min. Startle responses were normalized to the baseline startle amplitude measured in the initial 10 startle trials and PPI was calculated with the following formula:

$$PPI = 100\% - \left[ {\frac{baseline\;startle\;amplitude}{{startle\;prepulse\;trial}} \times 100} \right]$$

### Magnetic resonance imaging

A small-animal 4.7 T MRI scanner (BioSpec, Bruker BioSpin MRI GmbH, Ettlingen—Germany) equipped with a transmit and receive mouse body coil was used for image acquisition performed 5 weeks after the last administration. Animals were anesthetized with isoflurane (1.5–2%) prior and during the procedure. T1-weighted brain imaging was performed with a FLASH sequence (TR/TE/Flip = 50.5 ms/2.4 ms/30°) using an axial orientation and a spatial resolution of 0.18 × 0.18 × 0.7 mm. Quantitative image evaluation was performed blinded using manually defined regions of interest around the deep cerebellar nuclei (DCN) and the brain stem on each hemisphere as described previously^63^. The DCN / brain-stem signal intensity ratio was calculated by dividing the mean signal of the DCN by that of the brain stem.

### Synaptosome preparation and detergent treatment

Synaptosomes were prepared from six animals per time point and group with the exception of the 365 days p.i. time point where only three animals were available for synaptosomal preparation. Crude synaptosomes are prepared as previously described^64,65^. Briefly, one brain hemisphere of three animals each were pooled for homogenization in 30 ml sucrose buffer (320 mM sucrose, 5 mM HEPES pH 7.4) using a motor-driven glass-teflon homogenizer. Brain homogenates were centrifuged 10 min at 1000* g*, the resulting supernatant was transferred to a new tube and re-centrifuged for 15 min at 15,000* g*. The crude synaptosomal pellet was collected avoiding the brown mitochondrial pellet and snap frozen in liquid nitrogen and stored at − 80 °C. Synaptosomes were adjusted to 10 mg/ml protein concentration and aliquots of 500 µg synaptosomes were incubated with Triton- × 100 (3 g/g) or SDS (0.5% v/v) or an equal volume of buffer as a control for 30 min at 4 °C. Afterwards, samples were centrifuged for 5 min at 15,000*g*, the pellets were washed with 5 mM HEPES pH 7.4 and were re-centrifuged. The final pellet as well as the combined supernatants (extract and wash) were subjected to Gd analysis.

### Size exclusion chromatograpy (SEC) coupled to ICP-MS

The determination of the Gd species in the soluble extracts was performed using a Superdex 200 10/300 GL column (GE Healthcare Life Sciences) and an Agilent 1290 HPLC system coupled to an ICP-MS (Agilent 7900) as previously described^21^.

### Immunohistology LA-ICP-MS

Staining of frozen brain sections were performed as described^66^ with modifications for indirect detection using secondary metal-tagged antibodies. Briefly, sections were fixed in cold acetone (8 min. at − 20 °C), washed with PBS and blocked with 3% BSA for 45 min at room temperature prior to antibody incubation. Primary antibody directed against aggrecan (Merck Millipore #ab1031, 1:500) was incubated over night at 4 °C. Samples were washed 3× with PBS and subsequently incubated with appropriate metal-tagged secondary antibody (175Lu, Fluidigm #317002G) for 1 h at room temperature. Following washing with 0,005% Triton-X and PBS, nuclei were stained with the Cell-ID™ Intercalator-192/193Ir (Fluidigm, #201192A, 1:2000, 30 min). After washing with ddH2O, LA-ICP-MS samples were air-dried.

The distribution of elements was analyzed using an inductively coupled mass spectrometer (Agilent 7900) coupled to a laser ablation system (NWR 213, New Wave Research). Laser ablation of sections from samples was performed in continuous-line ablation mode with a laser frequency of 20 Hz. For larger specimen, a rectangular spot of 50 × 40 µm at a scan speed of 100 µm/s was used and for high resolution LA-ICP-MS, small regions of interest (1 mm^2^) were ablated with a circular spot size of 5 µm at a scan speed of 40 µm/s. The ablated tissue was transported into the ICP-MS by a constant stream of helium, 158Gd and the metal isotope of the secondary antibody 175Lu as well as 192Ir were measured in each ablated spot generating an intensity map of Gd, perineuronal net and nuclei throughout the region of interest. Gd was quantified by calibration using gelatin standards of known Gd concentration. Data processing to create elemental maps was performed using MassImager 3.17 software, which was developed and kindly provided by U. Karst and R. Schmid from the University of Muenster.

### Statistical evaluation

Data and statistical analysis were performed using Prism8.0.2 (GraphPad Software). After confirming normal distribution of acquired data (Shapiro–Wilk test), differences in the mean values between control and GBCA treated animals were compared using either ordinary 1‐way analysis of variance (ANOVA) with type I error protection by Dunnett (against control tests) or 2-way ANOVA with two between-subject factors (treatment and pulse intensity/time/…). For post-hoc comparison Dunnett`s correction for multiple comparison was used (splitted by pule intensity/time/…). A statistically significant difference between groups was defined as *p* < 0.05. Asterisks indicate significance at *p < 0.05 and **p < 0.01 throughout. Further specifications of particular statistics per assay are given in the supplementary information. All data represent mean ± SEM (Fig. 2, Fig. 3, Supplementary Figs. 1–3) or mean ± SD (Fig. 4).

Description of the remaining behavioral test (gait analysis, indirect calorimetry, and the cognitive test (operant conditioning using an operant wall)) as well as protocols for RNA sequencing, immunohistology and stereological analysis are given in the supplements.

### Ethical statement

All animal experiments presented here were approved by local ethical boards of the District Government of Lower Franconia, Bavaria, Germany (Approval No. DMS2532-2-397), and were conducted according to local, NIH, and ARRIVE guidelines^61^.

## Supplementary information

Supplementary Information 1.

## Data Availability

Data supporting the findings of this manuscript are available from the corresponding author upon reasonable request.

## Abbreviations

ANOVA: Analysis of variance
ASR: Acoustic startle reaction
BW: Bodyweight
Ca: Calcium
DCN: Deep cerebellar nuclei
DNA: Desoxyribonucleic acid
ECM: Extracellular matrix
FDA: Food and Drug Administration
GABA: Gamma-aminobutyric acid
GBCA: Gadolinium-based contrast agents
Gd: Gadolinium
LA-ICP-MS: Laser ablation inductively coupled plasma- mass spectrometry
MRI: Magnetic resonance imaging
LoQ: Limit of quantification
NSF: Nephrogenic systemic fibrosis
p.i.: Post injection
PNN: Perineuronal nets
PPI: Pre-pulse inhibition
SD: Standard deviation
SDS: Sodium dodecyl sulfate
SEC: Size exclusion chromatography
SEM: Standard error of the mean
SI: Signal intensity
