# Supplementary material for: Comprehensive phenotyping revealed transient startle response reduction and histopathological gadolinium localization to perineuronal nets after gadodiamide administration in rats
Source: Sci Rep. 2020 Dec 28;10:22385. doi: 10.1038/s41598-020-79374-z (PMC7769977; doi:10.1038/s41598-020-79374-z)
Supplement: Supplementary file 1 — Supplementary Information 1. [file 41598_2020_79374_MOESM1_ESM.pdf]

Supplementary Information

Comprehensive phenotyping revealed transient startle response reduction and histopathological gadolinium localization to perineuronal nets after gadodiamide administration in rats

Authors
Johanna Habermeyer[1], Janina Boyken[2], Julia Harrer[1], Fabio Canneva[1], Veronika Ratz[1], Sandra Moceri[1], Jakob Admard[3], Nicolas Casadei[3,4], Gregor Jost[2], Tobias Bäuerle[5], Thomas Frenzel[2], Christoph Schmitz[6], Gunnar Schütz[2], Hubertus Pietsch[2], Stephan von Hörsten[1]*

[1]Department of Experimental Therapy, University Hospital Erlangen and Preclinical Experimental Animal Center, Friedrich-Alexander-University Erlangen-Nuremberg, Germany.
[2]Bayer AG, MR & CT Contrast Media Research, Muellerstrasse 178, 13353, Berlin, Germany.
[3]Institute of Medical Genetics and Applied Genomics, University of Tuebingen, Tuebingen, Germany.
[4]DFG NGS Competence Center Tuebingen, 72076 Tuebingen, Germany
[5]Department of. Radiology, University Hospital Erlangen and Preclinical Imaging Center Erlangen (PIPE), Friedrich-Alexander-University Erlangen-Nuremberg (FAU), Germany
[6]Department of Neuroanatomy, Ludwig-Maximilians-University, Munich, Germany.

***Corresponding Author:**
Dr. Stephan von Hörsten, M.D.
Friedrich-Alexander-Universität Erlangen-Nürnberg (FAU)
Preclinical Experimental Center (PETZ)
Universitätsklinikum Erlangen (UKEr)
Dept. Experimental Therapy
Palmsanlage 5
91054 Erlangen
Germany
Office: +49 9131 85 23504
Fax: +49 9131 85 23502
Email: stephan.v.hoersten@fau.de
orcid.org/0000-0001-6409-0664

Supplementary methods

Animal Housing

Animals were housed under standard laboratory conditions (22 - 25 °C), with a constant 12 h light/12 h dark cycle, in stable social groups of 4 animals per cage and *ad libitum* access to water and food (standard rodent diet: SSniff, Soest - Germany). Experimental groups were splitted in cohorts of n = 4 animals per GBCA group and behavioral testing was shifted for 2 weeks in between each cohort to maintain optimal housekeeping, handling and experimental conditions.

OpenField test

To measure spontaneous locomotion as well as generalized anxiety-like behavior in a novel environment a classic open field (OF) paradigm was applied. Behavioral testing started by placing the rat in the middle of the OF and was stopped after 20 min of free exploration of the arena. Rat behavior was recorded with a video installed above the arenas. The video files were analyzed off-line using the Ethovision software (Noldus Information Technology, Wageningen – Netherlands). For analysis each arena of the OF was virtually subdivided into five areas (four corners and one center) allowing the determination of parameters attributed to anxiety-like behavior (time spent in center, time spent in corners). Grooming and rearing behavior were manually assessed, analyzing always two animals in parallel. More parameters attributed to exploratory behavior including distance moved and speed were automatically quantified by the software.

RotaRod test

For the assessment of fore- and hind-limb motor function and balance, an accelerating RotaRod system for rats (TSE-Systems GmbH, Bad Homburg - Germany) was used. The apparatus consisted of a base platform and a rotating rod with a non-skid surface. All animals of one cage were measured simultaneously. When operated in the acceleration modus, the rotor accelerates from 4 to 40 rpm in a period of 5 min.

Before experimental testing, animals performed four training sessions per day on the RotaRod with inter-trial intervals of around 20 minutes for three consecutive days at constant speed (12 rpm). During training sessions, the animals had to stay on the rod for a cutoff time of 180 s. Animals that fell off the rod were immediately placed back until their cutoff time was reached, intending to reinforce a constant performance. On day 4, animals performed the accelerod test in two consecutive trials at accelerating speed (4-40 rpm within 300 s) with 30 min inter-trial interval which they spent within their home cages. Quantitative measures were done on the latency to falling off the rod.

Gait analysis

A quantitative assessment of the gait, footsteps and motor performance was carried out using the CatWalk XT system (Noldus Information Technology, Wageningen - Netherlands). The apparatus is composed of an enclosed alley-shaped walkway though which the rats voluntarily traverse a glass plate which builds the floor and is horizontally illuminated by a green light. Each contact of a paw with the surface of the glass plate scatters the green light creating an image of the footprint, that is then captured by a video-camera positioned below the alley. The test was done on two days with 2 sessions consisting of 5 complete runs for each animal per day. The recorded runs were analyzed with the Illuminated FootprintTM technology and the CatWalk 10.6 software in parallel and incomplete runs were deleted immediately. Complete runs were evaluated offline (green intensity threshold: 0.12) and compliant runs (animal walks straight ahead from start to end, without stopping or turning on the alley) were investigated for the following parameters:

Base of support:	average width between either the front paws or the hind paws

Stand: duration of contact of a paw with the glass plate

Stride Length: the distance between successive placements of the same paw

Indirect calorimetry

The PhenoMaster system for rats (TSE Systems GmbH, Bad Homburg - Germany) is a modular high throughput system where individual animal are continuously monitored in a home-cage-like environment. Based on a combination of weight sensors and indirect calorimetry, this device enables to simultaneously measure circadian pattern of several metabolic and behavioral parameters at a high temporal resolution. As parameters relevant to the investigation, food and water consumption as well as the respiratory exchange rate were monitored during a standard 72 h experimental period. Two dedicated weighing sensors per cage registered ad libitum water and food consumption. Continuous mode calorimetry allowed the simultaneous calculation of O_2 consumption, CO_2 production and respiratory exchange rate (RER = $V(CO_2)$ / $V(O_2)$; performed by TSE PhenoMaster Software, version 4.4.6.). All gas sensor pairs were calibrated with calibration gas mixtures before each test session. Animals were kept isolated from the cage mates for the entire observational period, in a room that was maintained in the identical holding conditions as the rest of the colony. Disturbance of the animals was kept to a minimum, with the exception of short daily visits necessary to ensure the proper functioning of the system. Data were collected automatically with a rate of 100 Hz, stored as a sum over 1 min intervals and analyzed with the TSE PhenoMaster software. System use and data processing followed the description given in Urbach et al., 2014[77].

Cognitive testing

Cognitive testing using an operant conditioning system were performed during the dark phase, using a customized automated operant conditioning apparatus (TSE Systems GmbH, Bad Homburg - Germany) operated by the IntelliMaze software package (v. 4.0.0.0; NewBehavior, Germany). Rats were food deprived for 24 hours before starting operant conditioning for reasons of motivation of the animals. On each testing day, rats were introduced to the experimental cages containing the Operant Wall apparatus one hour before the light was turned off. Experimental testing started three hours after dark onset and consisted of three sessions administered at 3 hours intervals (including a 5 minutes inter-session interval). Every first session of the night was preceded by a “default” session which signaled to the animals the activation of the system and start of the experimental procedure. Animals were required to press one out of two presented levers according to a certain rule depending on the experimental protocol to receive a food reward (45 mg Dustless Precision Pellets, BioServ). Each morning rats were removed from the testing cages, were weighted and returned to their home cages and social groups. Complementary food was given during the day to obtain a food intake restricted to 90% of the calculated daily intake (as derived from experimental observation of the animals in the PhenoMaster cages), considering the maximal amount of pellets the rats can earn during the night.

General default phase: constituted the “adaptation” phase of the first session of the night independent of the experimental protocol. It the experiment, and was designed to deliver one pellet every 10 min in the food-crib situated at the center of the system. Additional rewards could be earned by the animals by pressing either of two levers. After successfully earning 10 rewards by pressing the levers the session was automatically terminated and the first experimental session of the night started. This initial adaptive phase had no time-limitations, thus allowing each animal to explore the walls for the necessary time before reaching sufficient confidence to start interacting with the levers

Habituation (2 consecutive nights): this protocol was designed in order to train the animals to a stable and consistent performance using the levers, thus eliminating biases due to insufficient motivation. In each session of the habituation paradigm 1 pellet was delivered for every lever press performed by the animal, on either of the levers available, up to a maximum

of 100 pellets/session. Rats that reached a stable performance of more than 90 pellets earned/session were then included into further testing.
Cued alternation (4 consecutive nights): the "cued alternation" paradigm constitutes a simple protocol for the investigation of associative memory. For each session of this task 100 trials were given, during which one light stimulus was presented randomly directly above the right or the left lever: pressing the lever corresponding to the stimulus resulted in the delivery of a reward, while pressing the opposite lever did not. In either case, both levers were shortly retracted before the next trial started. Percentage of earned pellets/session and side preference were measured by the system.

Sample collection

For sample collection rats were deeply anesthetized with a mix of ketamine (100 mg/kg BW) and xylazine (4 mg/kg BW) by an intraperitoneal injection. As soon as no reflexes of the tail and hind paws were observable, a trans-cardiac perfusion with ice-cold perfusion buffer was then performed for 2 min. The whole brain was removed and further processed for either biochemical analysis or immunohistochemistry. Samples for histological analysis were fixed in 4% paraformaldehyde in 0.1 M phosphate buffer for 24 h, equilibrated in 30% sucrose for additional 48 –72 h and snap-frozen in -70°C isopentan for 50 s. Cryosections were cut at 10 µm for LA-experiments or 40 µm thickness for immunohistology along the coronal axis on a freezing microtome (Leica CM3050 S Research Cryostat: Leica Biosystems, Nussloch - Germany) and stored in anti-freezing solution at -20 °C.

RNA sequencing

Fresh frozen tissues taken from independent animals were used for investigating gene expression. The RNeasy mini kit, including on column RNase-Free DNase I treatment (both Qiagen), was used for total RNA isolation (RNA ≥ 200bases), according to the manufacturers´ protocol. RNA quality was determined by measuring 260/280 and 230/260 ratio absorbance ratio on a spectrophotometer (Nanodrop ND-1000; Peqlab), RNA concentration was measured using the Qubit Fluorometric Quantitation and RNA Broad-Range Assay (Thermo Fisher Scientific, Waltham, MA - USA) and RNA Integrity Number RIN using the Lab-on-a-Chip-System Bio-analyzer 2100 and the RNA 6000 Nano assay (Agilent, Böblingen - Germany). For library preparation, mRNA fraction was enriched using poly-A capture from 100 ng of total RNA using the NEBNext Poly(A) mRNA Magnetic Isolation Module (NEB). Next, mRNA libraries were prepared using the NEB Next Ultra II Directional RNA Library Prep Kit for Illumina (NEB) according to the manufacturer's instructions. Library molarity was determined by measuring the library size (approximately 400 bp) using the Bioanalyzer2100 with the High Sensitivity DNA assay and the library concentration (approximately 10 ng/µl) using Qubit Fluorometric Quantitation and dsDNA High sensitivity assay (Thermo Fisher Scientific, Waltham, MA, USA). The library was denaturated according to the manufacturers´ instructions and diluted to 280 pM. Libraries were sequenced as paired-end 50 bp reads on an Illumina NovaSeq6000 (Illumina, San Diego, CA - USA) with a sequencing depth of approximately 25million clusters per sample.
Read quality of RNA-seq data in fastq files was assessed using ngs-bits (v.2019_03), to identify sequencing cycles with low average quality, adaptor contamination, or repetitive sequences from PCR amplification. Reads were aligned using STAR v2.6.1cdobin[64] to the Ensembl Rnor_6.0 genome (GenBank: GCA_000001895) and alignment quality was analyzed using ngs-bits (v.2019_03) and visually inspected in the Integrative Genome Viewer (v2.4.19). Normalized read counts for all genes were obtained using Subread (v1.6.3) and edgeR (v3.24.3). Raw expression values are available for 32,882 genes in 62 samples. Raw gene expression was filtered by demanding a minimum expression value of 1 cpm (counts per million) in at least 5 samples. Filtered data contains expression values for 14,918 genes. The distribution of logarithmized cpm-normalized expression values shows similar characteristics over all samples. Based on the filtered data set, samples were investigated with respect to their pairwise similarity. Spearman's rank correlation coefficient was calculated for each pair of samples. A hierarchical clustering was performed on the resulting similarity values. A multidimensional scaling analysis was performed, which is a dimension reduction method and

used to compress and visualize essential information found in the expression data. Spatial distances between samples correspond to differences in sample properties and approximate the typical expression differences (log2 fold change) between samples. Differential gene expression analysis was conducted based on the filtered gene expression data set. A statistical model incorporating the group property of samples was tested by fitting a negative binomial distribution using a generalized linear model (GLM) approach. For each gene, gene expression fold changes (log2 fold change) were computed and a statistical test was performed to assess the significance, which is given as raw p-value and adjusted p-value (FDR, obtained by Benjamini-Hochberg procedure).

Immunohistology

Sections of the cerebellum containing the DCN were picked and washed three times in PBS, once in PBS-T (PBS with 0.5% Triton X-100), and again in PBS (30 min each) to remove the anti-freezing solution in which sections had been stored. If needed, a 20 min peroxidase block was performed in PBS containing 0.3% H_2O_2, followed by washing in PBS-T (3x 10 min). Unspecific antibody binding was blocked with 5% normal donkey serum (Jackson ImmunoResearch, Suffolk - UK) in PBS-T (1 h). Following primary antibodies were used and incubated for 24 to 48 h at 4°C: rabbit anti-aggrecan antibody (Merck Millipore #ab1031, Darmstadt - Germany), 1:500 diluted, mouse anti-NeuN antibody (Merck Millipore MAB377). After washing off unbound primary antibody (3x 30 min in PBS-T), the following secondary antibodies were applied for 1h (biotin-labeled) or 48 h (fluorescence-labeled): biotin-labelled donkey anti-rabbit antibody (Santa Cruz Biotechnology sc-2089, Dallas - USA), 1:500 diluted, Alexa647-labeled donkey anti-mouse antibody (Thermo (Life), A-31571), 1:400 diluted.
For immunohistochemistry, after washing with PBS-T (2x 10 min) and PBS (10 min), AB-reagent (Vector Laboratories, Burlingame, USA) was applied for 30 min, followed by the same washing procedure, after which staining was developed using the DAB peroxidase substrate kit (Vector Laboratories, Burlingame, USA) with Nickel chloride solution according to manufacturers´ instructions for 2 min, washed in tab water (5 min) and PBS (10 min). Immunofluorescence stained sections were washed in PBS-T (3x 30 min), incubated with DAPI (1:1000) for 3 min and washed with PBS-T (3x 30min). All sections were finally mounted on glass slides (ThermoFisher Scientific, Waltham - USA). Immunohistochemistry-slides were dried overnight, dehydrated in ascending ethanol solutions (70%, 90%, 95%, and 2x 100%), and cleared twice in xylol, before adding DPX mounting medium (Sigma-Aldrich, St. Louis - USA) and cover slip. Sections were analyzed on a Keyence BZ9000 Generation II microscope and images taken with the attached BZ II Generation II analyzer Version 2.1 software (Keyence Corp., Osaka - Japan). White balance and post-processing were performed in Adobe photoshop CC (version 2015.1.2; Adobe systems, San José - USA). Immunofluorescence-slides were shortly dried and cover-slipped with Mowiol-488.

Stereological analysis

Image acquisition and analysis was performed as previously described[64]. Briefly, of every 6th section (40 mm thick sections) spanning the entire DCN Z-stacks (1mmsteps) of 40x images of NeuN and DAPI staining were acquired on a spinning-disk confocal micro-scope (BX51WI, Olympus). Unbiased analysis of the number of NeuN-positive cells in the lateral cerebellar nucleus and remaining DCN was conducted by a blinded experimenter. The Optical Fractionator method was applied, as offered by Stereo Investigator software (MBF Bioscience, Wiliston, VT – USA). Regions of interest were drawn manually, and section thickness was measured at every unbiased counting site. The height of the counting sites was set to 20 µm with a 2 µm guard zone above and below. Base area of the counting frame size was 100 µm x 100 µm with an SRS grid size of 100 µm x 100 µm for the lateral cerebellar nucleus and an SRS grid size of 200 µm x 200 µm for the remaining cerebellar nuclei. Averaged 434 counting frames for the lateral cerebellar nucleus and 244 counting frames for the remaining DCN per animal were manually evaluated.

Gadolinium quantification by inductively coupled plasma mass spectrometry (ICP-MS)

Pellets were spiked with terbium as an internal standard (5 nmol/l final concentration), dried at 90°C and solubilized by subsequent pressurized digestion in 50 µl concentrated nitric acid and 30 µl hydrogen peroxide. Solutions were diluted in 1% nitric acid containing 0.01% Tx-100 and the Gd concentration determined by ICP-MS (Agilent 7900, Germany). The system was calibrated using 1-1000 nmol Gd/l standard solutions. The limit of quantification (LOQ) was 0.5 nmol/L. Gd concentrations are shown as subtracted/stacked values. Total Gd corresponds to the Gd concentration found in the buffer control. Values below LOQ were set to zero.

Gdbuffer = (Gdbuffer – GdTx100)* + (GdTx100 – GdSDS)** + GdSDS***

* Gd released by Tx-100

** Gd released by SDS

*** insoluble Gd

Western Blot analysis

Brain homogenates were generated by established protocols[81,82]. Briefly, one hemisphere of three animals each were pooled a subsequently homogenized in 30 ml 320 mM sucrose, 5 mM HEPES pH 7.4 with a glass-teflon homogenizer (9 strokes at 900 rpm). Protein concentrations were determined by bicinchoninic acid assay (Thermo Scientific). Equal amounts of lysates were separated by SDS-PAGE, transferred to a nitrocellulose membrane (Invitrolon, Life Technologies) and blocked with Odyssey blocking buffer (LI-COR, Lincoln, NE). Membranes were incubated overnight at 4 °C with an anti-aggrecan (1:500, Merck Millipore) and anti-SDHA (1:2000, Abcam) antibody and then washed before incubation with IRDye secondary antibodies (LI-COR) for 1 h at room temperature. Membranes were analyzed using an Odyssey Infrared Imaging System (LI-COR; Millennium Science, Surrey Hills - Australia).

Supplementary Results

The statistical analysis of the ASR was conducted using a step-by-step strategy, going from superordinated coherences modeling the complex design of the experiment to collapsed analyses to allow interpretation of the data in the biological context. All results are summarized in Supplementary Table 1, for reasons of clarity and comprehensibility only the relevant effects are described in the following text.
3-way ANOVAs were applied investigating the effect of the factors “GBCA administration”, “dose” and “time post injection” as a repeated measurement for each pulse intensity, respectively. The factor “time post injection” was highly significant for all analyzed pulse intensities (75 db: $F_{2,306} = 81.233$, $p < 0.0001$; 95 db: $F_{2,306} = 14.085$, $p < 0.0001$; 105 db: $F_{2,306} = 15.822$, $p < 0.0001$; 115 db: $F_{2,306} = 8.666$, $p < 0.0002$; 120 db: $F_{2,306} = 10.906$, $p < 0.0001$). Therefore, 3-way ANOVAs investigating the effect of the factors “GBCA administration”, “dose” and “pulse intensity” at the different time points were conducted separately. Here. a significant effect of the factor “GBCA administration” was revealed at 7 weeks p.i. ($F_{1,977} = 4.486$, $p = 0.034$) but not at 14 ($F_{1,812} = 0.946$, $p = 0.331$) or 30 weeks p.i ($F_{1,797} = 0.024$, $p = 0.878$).
Based on these higher level analyses a 2-way ANOVA with subsequent post-hoc testing was applied, to investigate the effect of the combined factor “GBCA administration and dose” on ASR across pulse intensities 7 weeks p.i.
2-way ANOVA revealed a significant effect of the factor “pulse intensity” ($F_{4,750} = 269.5$, $p < 0.0001$), since startle reaction of all animals increased with rising pulse intensities (Supplementary Figure 1a). The factor “GBCA administration and dose” had a significant effect ($F_{6,750} = 4.797$, $p < 0.0001$) on the startle reaction of the treated animals. Conducting post-hoc Dunnett´s multiple comparison test (split per pulse intensity) revealed a significant difference in startle reaction of animals treated with 0.6 and 1.8 mmol/kg BW gadodiamide at 115 dB (post-hoc Dunnett´s multiple comparison test split per pulse intensity: 0.6 mmol/kg BW: $p = 0.0345$; 1.8 mmol/kg BW: $p = 0.0306$) and 120 dB (post-hoc Dunnett´s multiple comparison test split per pulse intensity: 0.6 mmol/kg BW: $p = 0.0117$; 1.8 mmol/kg BW: $p = 0.0266$) compared to the control group.
Measurements of the pre-pulse inhibition were analyzed following the same statistical model as described for the ASR experiment. All results are summarized in Supplementary Table 2, for reasons of clarity and comprehensibility only the relevant effects are described in the following text. 3-way ANOVAs were conducted analyzing the effect of the factors “GBCA administration”, “dose” and “time post injection” as a repeated measurement for each pre-pulse intensity, respectively. The factor “time post injection” was highly significant for all analyzed pre-pulse intensities (72 db: $F_{2,302} = 3.834$, $p = 0.0227$; 76 db: $F_{2,302} = 24.245$, $p < 0.0001$; 80 db: $F_{2,302} = 34.749$, $p < 0.0001$; 84 db: $F_{2,302} = 34.258$, $p < 0.0001$;). Therefore, 3-way ANOVAs investigating the effect of the factors “GBCA administration”, “dose” and “pre-pulse intensity” at the different time points were applied separately. No significant effect of the factor “GBCA administration” was revealed at 7 weeks p.i. ($F_{1,772} = 2.835$, $p = 0.0926$), 14 weeks p.i. ($F_{1,648} = 0.959$, $p = 0.3277$) or 30 weeks p.i ($F_{1,636} = 1.036$, $p = 0.3091$).

Animals treated with 1.8 mmol/kg BW gadodiamide without any NSF-like skin lesions spend similar time grooming in the OpenField test compared with the control group (1-way ANOVA: $F_{2,46} = 0.7739$, $p = 0.4671$; Supplementary Figure 1g). 2-way ANOVA of repeated measures of the animals´ BW revealed no “time x GBCA administration” interaction ($F_{12,244} = 1.471$, $p = 0.1357$). The BW of all animals increased with the aging of the animals (factor “time”: $F_{1.341,163.6} = 1675$, $p < 0.0001$) as a result of the normal progressive weight gain observed under standard housing conditions (Supplementary Figure 1h). The parameter “GBCA administration” ($F_{6,122} = 0.8533$, $p = 0.5315$) had no significant effect on the BW of the animals.

The TSE PhenoMaster System was used to assess the circadian pattern of ingestion behavior as well as indirect calorimetric parameters in a home-cage environment. Overall water (Supplementary Figure 2a) and food intake (Supplementary Figure 2b) was analyzed by 1-way ANOVA, which revealed no effect of the GBCA administration (water intake: $F_{2,50} = 1.444$, $p = 0.2456$; food intake: $F_{2,50} = 2.173$, $p = 0.1245$). The respiratory exchange ratio followed a

circadian rhythm with enhanced quotients during the dark phase compared to the light phase (Supplementary Figure 2c). 2-way ANOVA of repeated measures revealed no “time x GBCA administration” interaction ($F_{280,7000} = 0.9028$, $p = 0.8730$) and no effect of the individual factor “GBCA administration” ($F_{2,50} = 0.1797$, $p = 0.8361$) but an influence of the factor “time” ($F_{14.79,739.7} = 28.44$, $p < 0.0001$) describing the circadian pattern.
To investigate cognitive performance, the animals were tested in an alternation cued task, in which they received a reward after pressing a lever on the same side as the light stimulus was presented. All three groups of animals were able to earn more than 90% of the pellets after 4 nights (Supplementary Figure 2d). 2-way ANOVA of repeated measures revealed no “time x GBCA administration” interaction ($F_{22,583} = 0.8392$, $p = 0.6768$). The administration of GBCA had no influence on the performance in this testing paradigm ($F_{2,53} = 0.6806$, $p = 0.5107$), while the learning of the animals over time resulted in a significant effect of the factor “number of testing sessions” ($F_{4.297,227.7} = 162.8$, $p < 0.0001$).

To investigate fore- and hind-limb motor coordination and balance, the RotaRod test was carried out. Animals of all three groups showed comparable performance (Supplementary Figure 2e) and no effect of GBCA administration (1-way ANOVA: $F_{2,56} = 0.2077$, $p = 0.8131$) was seen.

In line with published preclinical and clinical data, Gd accumulated in the region of the lateral cerebellar nucleus in animals that received gadodiamide but not gadobutrol (Supplementary Figure 3a). We further investigated the perineuronal nets as possible interaction partner for released Gd due to their ability to bind metal ions. The overall protein expression of aggrecan in full brain homogenates obtained 5 weeks p.i. was comparable (Supplementary Figure 3b).

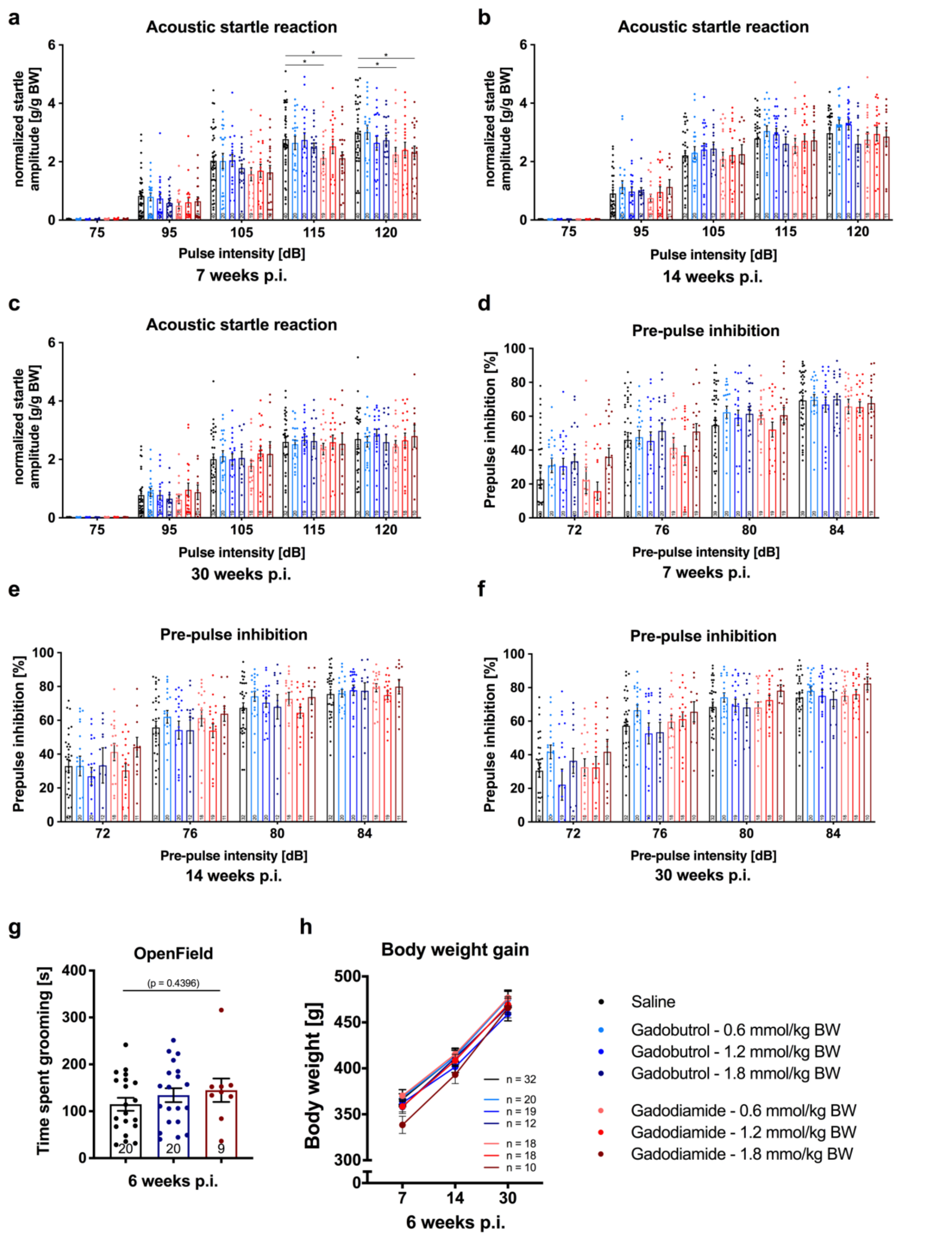

Supplementary Figure1

Acoustic startle reaction and pre-pulse inhibition of animals treated with GBCA as well as grooming behavior in the OpenField test and bodyweight of the animals. **a-c** Startle reaction to acoustic stimuli of different intensities measured 7, 14 and 30 weeks p.i. **d-f** Pre-pulse inhibition induced by increasing pre-pulse intensities 7, 14 and 30 weeks p.i. **g** Time spent grooming in the OpenField test, excluding animals with NSF-like skin lesions measured 5 weeks p.i. **h** Body weight gain in all groups of animals over time. Data represent mean ± SEM; n is indicated in the columns (a - g) or graph (h).

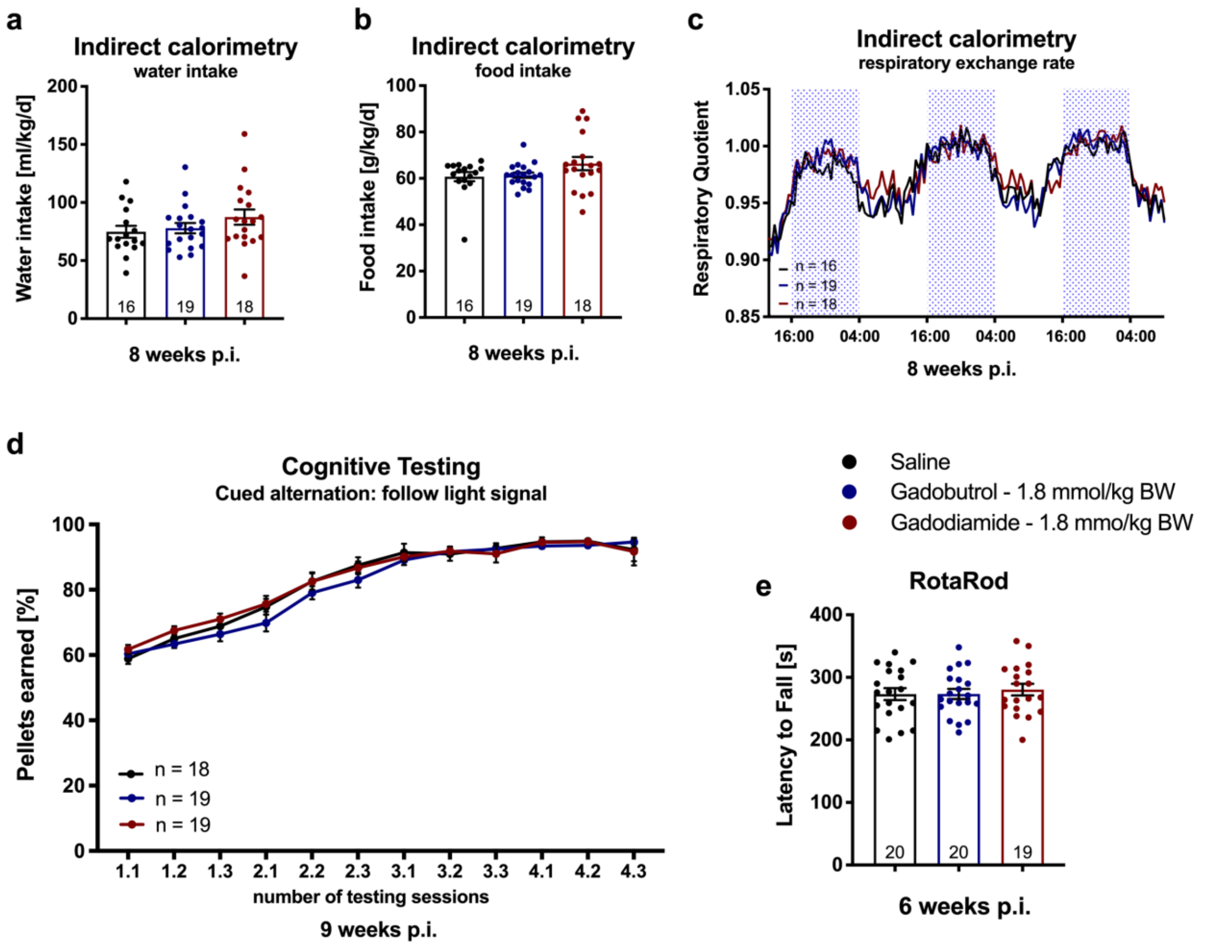

Supplementary Figure2

No alteration of the metabolic and cognitive status by administration of GBCA **a** Water intake, **b** food intake and **c** respiratory exchange rate over 24 h measured by indirect calorimetry using the automated PhenoMaster system 8 weeks p.i. **d** Alternation task learned by operant conditioning 9 weeks p.i. **e** Latency to fall off an accelerating rod 6 weeks p.i. Data represent mean ± SEM; n is indicated in the columns (a and b) or graphs (c and d).

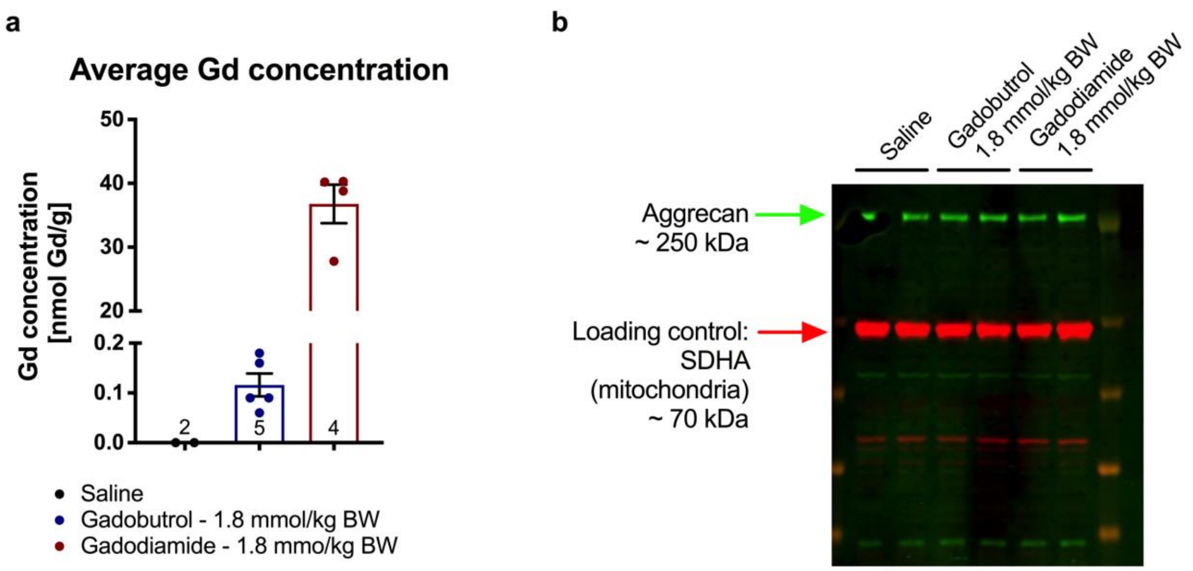

Supplementary Figure3

Gd concentration in the deep cerebellar nuclei and aggrecan expression in animals treated with GBCA **a** Average Gd concentration in nmol/g in the deep cerebellar nuclei at 11 weeks p.i. determined by LA-ICP-MS. **b** Western blot analysis of the protein expression of the perineuronal net extracellular matrix protein aggrecan (green) and mitochondrial protein SDHA (red) in full brain homogenates generated 5 weeks p.i. Data represent mean ± SEM; n is indicated in the columns.

Supplementary Table1: Summary of statistical analyses of ASR experiment

3way ANOVA for repeated measurement: ASR to **75 db** across time			
	DF	F	p
GBCA administration	1	15,222	0,0001
Dose	3	1,469	0,2252
GBCA administration * Dose	3	3,208	0,0248
**Time**	**2**	**81,233**	**< 0,0001**
Time * GBCA administration	2	4,033	0,0187
Time * Dose	6	2,009	0,0643
Time * GBCA administration * Dose	6	1,88	0,0839
3way ANOVA for repeated measurement: ASR to **95 db** across time			
	DF	F	p
GBCA administration	1	0,266	0,6065
Dose	3	0,1	0,96
GBCA administration * Dose	3	0,796	0,4977
**Time**	**2**	**14,085**	**< 0,0001**
Time * GBCA administration	2	1,168	0,3122
Time * Dose	6	1,069	0,3811
Time * GBCA administration * Dose	6	0,487	0,8177
3way ANOVA for repeated measurement: ASR to **105 db** across time			
	DF	F	p
GBCA administration	1	1,141	0,287
Dose	3	0,155	0,9261
GBCA administration * Dose	3	0,309	0,8188
**Time**	**2**	**15,822**	**< 0,0001**
Time * GBCA administration	2	1,607	0,2022
Time * Dose	6	1,051	0,3921
Time * GBCA administration * Dose	6	0,683	0,6637
3way ANOVA for repeated measurement: ASR to **115 db** across time			
	DF	F	p
GBCA administration	1	1,299	0,2561
Dose	3	0,557	0,644
GBCA administration * Dose	3	0,395	0,7571
**Time**	**2**	**8,666**	**0,0002**
Time * GBCA administration	2	0,687	0,504
Time * Dose	6	1,343	0,2376
Time * GBCA administration * Dose	6	0,379	0,8924
3way ANOVA for repeated measurement: ASR to **120 db** across time			
	DF	F	p
GBCA administration	1	1,187	0,2777
Dose	3	0,485	0,6929
GBCA administration * Dose	3	0,653	0,5821
**Time**	**2**	**10,906**	**< 0,0001**
Time * GBCA administration	2	2,227	0,1096
Time * Dose	6	3,213	0,0045
Time * GBCA administration * Dose	6	0,577	0,7483
3way ANOVA: ASR to different pulse intensities **7 weeks p.i.**			
	DF	F	p
**GBCA administration**	**1**	**4,486**	**0,034**
Dose	3	2,557	0,054
GBCA administration * Dose	3	1,005	0,39
→ significant effect of "GBCA administration": subsequent 2way ANOVA (with combined "GBCA administration and dose" factor			
2way ANOVA: ASR to different pulse intensities **7 weeks p.i.** ("GBCA administration and dose" combined)			
	DF	F	p
**GBCA administration and dose**	**6**	**4,797**	**< 0,0001**
Pulse intensity	4	269,5	< 0,0001
GBCA administration and dose * Pulse intensity	24	0,6281	0,9165
3way ANOVA: ASR to different pulse intensities **14 weeks p.i.**			
	DF	F	p
GBCA administration	1	0,946	0,331
Dose	3	0,123	0,946
GBCA administration * Dose	3	0,693	0,557
→ no significant effects: no subsequent 2-way ANOVA			
3way ANOVA: ASR to different pulse intensities **30 weeks p.i.**			
	DF	F	p
GBCA administration	1	0,024	0,878
Dose	3	0,378	0,769
GBCA administration * Dose	3	0,341	0,795
→ no significant effects: no subsequent 2-way ANOVA			

DF: degrees of freedom

Supplementary Table2: Summary of statistical analyses of PPI experiment

	DF	F	p
3way ANOVA for repeated measurement: PPI to **72 db** across time			
	DF	F	p
GBCA administration	1	0,136	0,7127
Dose	3	2,631	0,0522
GBCA administration * Dose	3	0,389	0,7609
**Time**	**2**	**3,834**	**0,0227**
Time * GBCA administration	2	2,091	0,1254
Time * Dose	6	0,575	0,7504
Time * GBCA administration * Dose	6	1,085	0,3714
3way ANOVA for repeated measurement: PPI to **76 db** across time			
	DF	F	p
GBCA administration	1	0,211	0,6464
Dose	3	0,923	0,4313
GBCA administration * Dose	3	0,731	0,535
**Time**	**2**	**24,245**	**< 0,0001**
Time * GBCA administration	2	0,966	0,3819
Time * Dose	6	0,922	0,4795
Time * GBCA administration * Dose	6	0,504	0,8051
3way ANOVA for repeated measurement: PPI to **80 db** across time			
	DF	F	p
GBCA administration	1	0,011	0,9157
Dose	3	1,374	0,2528
GBCA administration * Dose	3	0,703	0,5515
**Time**	**2**	**34,749**	**< 0,0001**
Time * GBCA administration	2	0,323	0,7243
Time * Dose	6	0,512	0,7993
Time * GBCA administration * Dose	6	0,513	0,7983
3way ANOVA for repeated measurement: PPI to **84 db** across time			
	DF	F	p
GBCA administration	1	0,058	0,8104
Dose	3	0,319	0,8113
GBCA administration * Dose	3	0,385	0,7643
**Time**	**2**	**34,258**	**< 0,0001**
Time * GBCA administration	2	0,683	0,5061
Time * Dose	6	0,593	0,7356
Time * GBCA administration * Dose	6	0,86	0,5248
3way ANOVA: PPI to different pre-pulse intensities **7 weeks p.i.**			
	DF	F	p
GBCA administration	1	2,835	0,0926
Dose	3	2,226	0,0838
GBCA administration * Dose	3	0,99	0,397
→ no significant effects: no subsequent 2-way ANOVA			
3way ANOVA: PPI to different pre-pulse intensities **14 weeks p.i.**			
	DF	F	p
GBCA administration	1	0,959	0,3277
Dose	3	2,04	0,1071
GBCA administration * Dose	3	0,676	0,5671
→ no significant effects: no subsequent 2-way ANOVA3			
3way ANOVA: PPI to different pre-pulse intensities **30 weeks p.i.**			
	DF	F	p
GBCA administration	1	1,036	0,3091
Dose	3	1,61	0,1857
GBCA administration * Dose	3	2,297	0,0766
→ no significant effects: no subsequent 2-way ANOVA			

DF: degrees of freedom
